# The Beneficial Effects of *Lactobacillus plantarum* PS128 on High-Intensity, Exercise-Induced Oxidative Stress, Inflammation, and Performance in Triathletes

**DOI:** 10.3390/nu11020353

**Published:** 2019-02-07

**Authors:** Wen-Ching Huang, Chen-Chan Wei, Chi-Chang Huang, Wen-Lin Chen, Hui-Yu Huang

**Affiliations:** 1 Department of Exercise and Health Science, National Taipei University of Nursing and Health Sciences, Taipei 11219, Taiwan; wenching@ntunhs.edu.tw; 2 Department of Aquatic Sport, University of Taipei, Taipei 11153, Taiwan; tom911072@gmail.com; 3 Graduate Institute of Sports Science, National Taiwan Sport University, Taoyuan 33301, Taiwan; john5523@mail.ntsu.edu.tw; 4 Department of Food Science, Nutrition, and Nutraceutical Biotechnology, Shih Chien University, Taipei 10462, Taiwan; maggieh323@hotmail.com; 5 Graduate Institute of Metabolism and Obesity Sciences, Taipei Medical University, Taipei 11031, Taiwan

**Keywords:** *L. plantarum* PS128, performance, inflammation, oxidative stress, triathlon

## Abstract

A triathlon, which consists of swimming, bicycling, and running, is a high-intensity and long-term form of exercise that can cause injuries such as muscular damage, inflammation, oxidative stress, and energy imbalance. Probiotics are thought to play an important role in disease incidence, health promotion, and nutrient metabolism, but only a few studies have focused on physiological adaptations to exercise in sports science. Previous studies indicated that *Lactobacillus* supplementation could improve oxidative stress and inflammatory responses. We investigate the effects of *Lactobacillus plantarum* PS128 supplementation on triathletes for possible physiological adaptation. The triathletes were assigned to one of two groups with different exercise intensity stimulations with different time-points to investigate the effects of body compositions, inflammation, oxidative stress, performance, fatigue, and injury-related biochemical indices. *L. plantarum* PS128 supplementation, combined with training, can significantly alleviate oxidative stress (such as creatine kinase, Thioredoxin, and Myeloperoxidase indices) after a triathlon (*p* < 0.05). This effect is possibly regulated by a 6–13% decrease of indicated pro-inflammation (TNF-α, IL-6, and IL-8) cytokines (*p* < 0.05) and 55% increase of anti-inflammation (IL-10) cytokines (*p* < 0.05) after intensive exercise stimulation. In addition, *L. plantarum* PS128 can also substantially increase 24–69% of plasma-branched amino acids (*p* < 0.05) and elevate exercise performance, as compared to the placebo group (*p* < 0.05). In conclusion, *L. plantarum* PS128 may be a potential ergogenic aid for better training management, physiological adaptations to exercise, and health promotion.

## 1. Introduction

The triathlon, a combination of swimming, cycling, and running exercises, originated in 1972 in San Diego, California with a non-positive competition. Participants were required to complete 3.8 km of swimming, 180.0 km of cycling, and 42.2 km of running within one day. The event tested the athletes’ fitness, speed, and athletic skills. In 2006, the Asian Games in Doha, Qatar included a triathlon. The triathletes had to complete 1.5 km of swimming, 40.0 km of biking, and 10.0 km of running (51.5 km in total) in line with independent and continuous procedures for the official international competition schedule [1].

A triathlon can induce many sports injuries because it is a form of high-intensity and long-term sport. Triathlons have been proven to cause muscular injuries and fatigue [2]. Most muscle damage caused by exercise is due to an increase in exercise-related oxidative stress, which leads to a decline in skeletal function [3]. A previous study also addressed two important aspects related to causing exercise-induced muscle damage (EIMD)—namely, mechanical and inflammatory stress [4]. Intensive endurance exercise also increases oxygen consumption, ischemia-reperfusion injury, white blood cell activation, inflammation, and reactive oxygen species (ROS) production [5,6].

According to the definition of probiotics by the Food and Agriculture Organization (FAO) of the United Nations and the World Health Organization (WHO), live microorganisms administered in adequate amounts can confer a health benefit to the host [7]. Many microorganisms are involved in a host’s metabolism and can metabolize food into nutrients and energy for the host’s utilization. Health benefits have mainly been demonstrated for specific probiotic strains of the following genera: *Lactobacillus*, *Bifidobacterium*, *Saccharomyces*, *Enterococcus*, *Streptococcus*, *Pediococcus*, *Leuconostoc*, and *Bacillus*, *Escherichia coli* [8]. Lactic acid bacteria are a probiotic in the human body and belong to the Gram-positive and facultative group of anaerobic bacteria. Many studies have shown that *Lactobacillus* exerts various biological effects, such as improving Type 2 diabetes mellitus [9], reducing the incidence of obesity-related metabolic diseases [10], regulating blood pressure [11], and exerting anti-inflammatory and anti-oxidation effects [12,13].

Probiotic studies in sport sciences have been quite limited in terms of revealing potential applications and benefits. In our previous studies, we reported that the *Lactobacillus plantarum* TWK10 could possibly improve exercise performance via energy regulation [14] and muscular adaptation [15] with clinical and animal trials. In addition, *Lactobacillus helveticus* Lafti L10 can be a beneficial nutritional supplement, not only for the reduction of upper respiratory tract illness (URTI) length, but also for the improvement of systemic humoral and mucosal immune responses in elite athletes [16,17]. *Lactococcus lactis* JCM 5805, administered via heat-killed supplementation, relieves URTI morbidity and symptoms via dendritic cell activation without affecting muscle damage and stress markers in athletes [18].

*Lactobacillus plantarum* PS128 was previously reported to yield beneficial improvement to anxiety-like behaviors, and it may be helpful for ameliorating neuropsychiatric disorders via possible inflammatory cytokines and neurotransmitter modulation [19]. Therefore, we focus on the possible effects of *L. plantarum* PS128 on triathletes from a sport nutrient viewpoint. Triathlon athletes regularly undergo intensive training and competition. Physiological stress and adaptations, especially for athletes, is therefore an interesting topic to study to determine whether *L. plantarum* PS128 can provide physiological benefits.

## 2. Materials and Methods

### 2.1. Lactic Acid Bacteria

*Lactobacillus plantarum* PS128 was provided by Professor Ying Chieh Tsai at Yang Ming University (Taipei, Country) and was cultivated and produced by Synbio Tech Inc. (Kaohsiung, Taiwan). The lyophilized *L. plantarum* PS128 powder was encapsulated as capsules by Prince Pharmaceutical Co., Ltd (New Taipei City, Taiwan). Each capsule included 300 mg of lyophilized bacteria powder, equivalent to 1.5 × 10^10^ colony-forming units (CFU), and 100 mg excipient of microcrystalline cellulose. Placebo capsules were also filled with 400 mg excipient of microcrystalline cellulose. The capsules were supplemented twice per day (one capsule/time), after training and before sleeping (3 × 10^10^ CFU/day). The subjects were required to take the capsules after training and before sleeping twice per day for the whole of the supplementation duration.

### 2.2. Subjects

In Study I, we recruited 18 subjects from triathlon teams at Taipei City University and randomly divided them into two groups: a placebo group (PG; *n* = 9) and a *L. plantarum* group (LG; *n* = 9). Study II also recruited 16 subjects (*n* = 8 for both groups) from the same university for different experimental designs. The athletes in studies I & II were different people because the training programs and period were already well-arranged for competition strategies and goals. Therefore, we separated our study into different parts to conduct our investigation. Table 1 shows the basic characteristics of the triathletes in current studies.

### 2.3. Experimental Design

This study was based on a double-blind experimental design to realize the possible effects of *L. plantarum* PS128 supplementation. Subjects were asked to maintain their regular lifestyle 24 hours before any experimental test and to avoid any strenuous exercise, staying up late, engaging in smoking, and consuming alcoholic beverages. While the supplements were taken, the subjects were prohibited from consuming probiotics, prebiotics, fermented products (yogurt or other foods), vitamins, materials, herbal extracts, and antibiotics to avoid unnecessary interference during the experimental periods. The participants were also asked to provide written informed consent before participating in the study. The study was reviewed and approved by the Institutional Review Board of Taipei City University (Taipei, Taiwan; No. 2015-025). 

In Study I, the 18 subjects were arranged with eight weeks of programmed training during the preparation period; the last four weeks included indicated supplementation. The subjects were challenged with a sprint triathlon (swimming 750 m, biking 20 km, and running 5 km). Blood sampling was done at basal level (before supplementation), immediately after exercise (AfterEx) and after 3 h of rest (3hRest) for biochemical and cytokine analysis.

**Figure 1:**
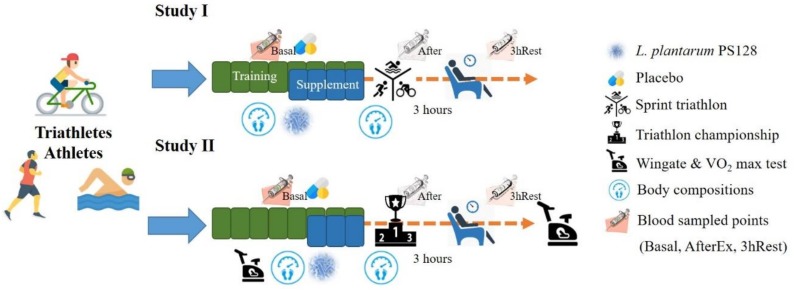
Experimental scheme.

In Study II, the protocol was similar; however, the three weeks of supplementation included a specialized training period. The aerobic and anaerobic capacities of the athletes were also measured before and after supplementation. Instead of a sprint triathlon, the official competition of a triathlon championship provides much higher exercise stimulation for the study of physiological adaptation. Body compositions were also evaluated before and after supplementation in both studies. The detailed experimental procedure is illustrated in Figure 1. For the dietary records before exercise (sprint triathlon and triathlon championship), the athletes were provided standardized food that amounted to about 595 kcal, including 24 g proteins, 16 g fat, and 90 g carbohydrates. In addition, athletes were recommended to uptake 30–40 grams of carbohydrates and 500–1000 ml water/hour for sufficient energy supply during the triathlon championship.

### 2.4. Body Compositions

Body composition was measured using a dual-energy X-ray absorptiometer (DEXA) (Dual-Energy X-ray Absorptiometry, GE Lunar, Madison, WI, USA) as a non-invasive test instrument. Participants were advised not to wear any clothing or accessories with metal zippers or metal buttons before testing. The participants lay in the scanning range of the DEXA. The DEXA test was started after the platform was entered; the height, weight, date of birth, and sex of the participants were entered separately. The detection time was roughly 10 minutes. The DEXA scans the body with X-rays of different energies. After X-rays of different energies penetrate the bone and soft tissue, the X-ray absorption or attenuation of the different tissues was calculated by the built-in formula of the machine.

### 2.5. Triathlon Championship and Sprint Triathlon

The triathlon championship included three competitions: 1.5 km of swimming, 40.0 km of biking, and 10.0 km of running, where the top three competition times and the total time of the game was provided by the conference. The sprint triathlon was only half the distance of the full triathlon.

### 2.6. Blood and Urine Sampling

In Studies I and II, blood samples were collected from the subjects at the AfterEx and 3hRest points after the exercise challenge, and the samples were collected into collection tubes containing anticoagulant EDTA and Heparin. The plasma and serum without anticoagulant were centrifuged at 3000× *g* for 10 minutes. The plasma and serum were stored at −70 °C for further analysis. In Study II, the collected urine also collected at the basal, AfterEx, and 3hRest0 points and were centrifuged at 1500 g for 15 minutes and filtered through a filter with a pore size of 0.45 μm. The plasma, serum, and urine were stored at −70 °C until further analysis.

### 2.7. Biochemical Variables

Lactic acid, ammonia, lactate Dehydrogenase (LDH), creatine kinase (CK), and myoglobin in the serum were detected using a SIEMENS ADVIA 1800 automatic biochemical analyzer (SIEMENS, Erlangen, Germany). Free fatty acids (BioVision, K612-100, San Francisco, USA) and protein carbonyl (Cayman, 10005020, Ann Arbor, Michigan, USA) in the serum were quantified using a colorimetric kit and enzyme-linked immunosorbent assay (ELISA) methods.

### 2.8. Amino Acid Analysis

A Hitachi L-8900 Amino Acid Analyzer (Hitachi, Japan) was used to detect and quantify alanine, phenylalanine, cysteine, aspartic acid, asparagine, glutamic acid, glutamine, glycine, histidine, leucine, isoleucine, lysine, proline, arginine, serine, threonine, valine, tryptophan, tyrosine, and methionine in the plasma samples.

### 2.9. Inflammatory Cytokines and Markers Analysis

The inflammation-associated serum cytokines TNF-α, IFN-γ, IL-2, IL-4, IL-6, IL-10 (BioLegend, San Diego, CA, USA), and Myeloperoxidase (MPO; Abcam, Cambridge, MA, USA) were analyzed using colorimetric kits. Thioredoxin (TRX; Cloud-Clone Corp, SEA702Hu, USA) and Complement Component 5a (C5a; Cloud-Clone Corp, SEA388Hu, Houston, USA) in the urine were evaluated for the effects of exercise-induced inflammation, and also assessed with colorimetric kits. The procedures followed the instructions of the kits, and the findings were measured using an ELISA reader (BioTek, PowerWave XS2, Winooski, USA).

### 2.10. Anaerobic and Aerobic Capacities Analysis

Wingate and VO_2_ max were used to study the athletes’ anaerobic and aerobic capacities, respectively, after supplementation. Participants performed a 30 second Wingate anaerobic kinetic test on a stationary bicycle (Wattbike Pro, Nottingham, UK) to assess their anaerobic exercise capacity. All participants were given a sufficient warm-up period before the official test. The subjects were required to try their best on the bike for 30 seconds with timely encouragement after the end of the warm-up stage. During the 30 second test period, the bike recorded and analyzed the athletes’ number of laps, watts produced, peak anaerobic power (PP), mean power (MP), and fatigue index (FI), which were all described in a previous study [20]. The VO_2_ max endurance test was performed on a Cortex gas analyzer (Cortex Biophysik, Germany) and a stationary bicycle (Monark ergometer, Sweden). Participants were given a 3 minute warm-up period and took a break on the bicycle for 3 minutes prior to the test. The 85% VO_2_ max speed, adjusted by the individual VO_2_ max, was applied to individual subjects until they reached exhaustion in the endurance assessment [14]. The pre-tests and post-tests were performed before supplementation and after the championship triathlon, respectively, in Study II.

### 2.11. Statistics

All of the results were statistically analyzed with SPSS 18.0 (IBM, New York, USA) and tested using the repeated one-way analysis of variance (ANOVA), followed by Student’s paired and unpaired *t*-tests. The values were expressed as mean ± SEM, and the mean and standard error of each value were calculated using the complete raw data. The final numbers were rounded to the appropriate numerical presentation. The significant difference within and between groups was considered when *p* < 0.016 (0.05/3).

## 3. Results

### 3.1. Body Compositions Pre- and Post-Supplementation

**Figure 2:**
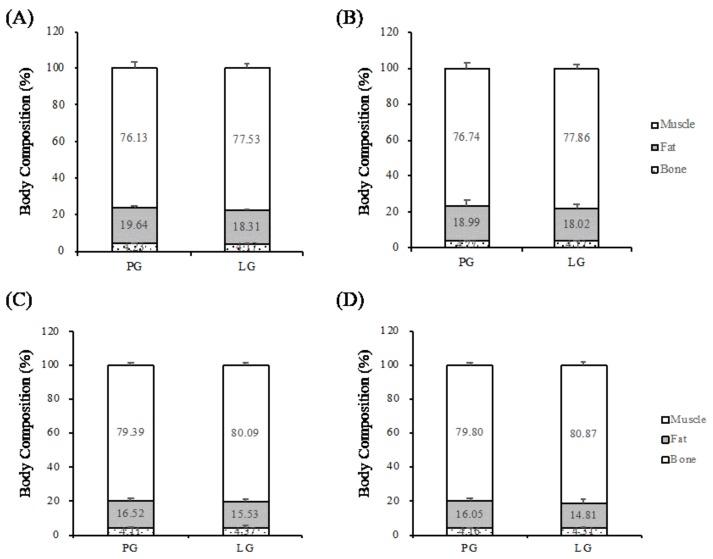
Body compositions between pre- and post-supplementation in the two studies. The data are represented as mean ± SEM. Study I included subjects (**A**) pre- and (**B**) post-supplementation with muscle, fat, and bone percentages (%) in the placebo group (PG) (*n* = 9) and *L. plantarum* group (LG) (*n* = 9). Study II included subjects (**C**) pre- and (**D**) post-supplementation with muscle, fat, and bone percentages (%) in the PG (*n* = 8) and LG (*n* = 8). *p* < 0.05 was considered to be a statistically significant difference within and between the groups.

The body compositions of the subjects were analyzed using DEXA. There was no significant difference in fat-free mass, fat mass, or bond mass between the PG and LG groups pre- and post-supplementation (*p* ≥ 0.05) (Figure 2).

### 3.2. Effects of L. plantarum PS128 on Fatigue and Injury-Related Biochemical Indices

In Study I (Table 2), LDH, ammonia, lactate, and FFA did not exhibit show significant differences between groups at the AfterEx and 3hRest points after the sprint triathlon challenge. The sprint triathlon stimulation induced a significant physiological increase in LDH, ammonia, lactate, and FFA indices compared with the basal measurements. After 3 h of recovery (3hRest), the ammonia and lactate indices immediately and significantly (*p* < 0.05) decreased, compared with AfterEx. In the much tougher exercise challenge with the competitive triathlon championship (Study II; Table 3), we noted a similar result for the CK, LDH, myoglobin, and FFA indices; there was no significant difference between the groups at the AfterEx point. However, the LG group exhibited a significant decrease (*p* < 0.05) in CK index during the recovery phase (3hRest) compared with the PG group.

### 3.3. Effects of L. plantarum PS128 on Inflammation Cytokines after Intense Exercise

In Table 4 and Table 5, the effects of *L. plantarum* PS128 on the inflammation cytokine profiles with intense exercise stimulation are listed. In Study I (Table 4), the TNF-α, IL-6, IL-8, and IL-10 cytokines were significantly higher at AfterEx than at the basal point in the PG; the *L. plantarum* PS128 supplementation (in the LG) significantly decreased intense exercise-induced inflammation cytokines, such as TNF-α, IL-6, and IL-8 (*p* < 0.05). At the recovery phase, IL-6 was still significantly lower in the LG than the PG (*p* < 0.05). In Study II (Table 5), the triathlon championship-induced inflammation cytokines, TNF-α, IL-6 and IL-10, were also significantly elevated in the PG, and there was a significant decrease in TNF-α and IL-6 cytokines with *L. plantarum* PS128 supplementation at AfterEx. IL-10 in the LG was significantly higher than in the PG. During the recovery phase, the LG also demonstrated a significant decrease in the TNF-α, IFN-γ, IL-6, and IL-8 cytokines compared with the PG (*p* < 0.05).

### 3.4. Effects of L. plantarum PS128 on Kidney Injury and MPO Level after Intense Exercise

**Figure 3:**
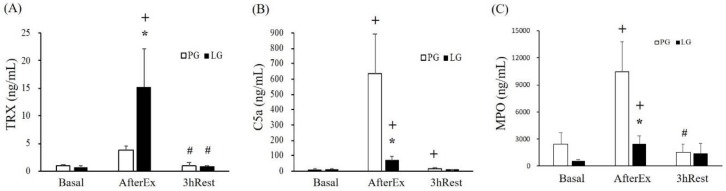
The profiles of kidney injury and inflammation-associated markers were collected and assessed from the participants in Study II who engaged in a championship triathlon intervention. (**A**) TRX (Thioredoxin), (**B**) C5a (Component 5a), and (**C**) MPO (Myeloperoxidase) were assessed at multiple time points included Basal, AfterEx, and 3hRest, and the data were represented as mean ± SEM. * indicates a significant difference between groups; ^+^ indicates a significant difference compared with the basal point within groups; and ^#^ indicates a significant difference compared with the AfterEx point within the groups. The significant difference within and between groups was considered when *p* < 0.016 (0.05/3).

The urine samples were further analyzed using the TRX and C5a markers. Figure 3A,B reveal that TRX and C5a were significantly increased in both groups at the AfterEx point. *L. plantarum* PS128 supplementation (LG) significantly increased and decreased the TRX and C5a indices compared with the PG. The substantial increase in MPO level as a function of intense exercise may be significantly ameliorated by *L. plantarum* PS128 supplementation (Figure 3C).

### 3.5. Effects of L. plantarum PS128 on Anaerobic and Aerobic Exercise Capacities

Anaerobic and aerobic exercise capacities were assessed after the triathlon championship using Wingate and 85% VO_2_ max, respectively. The data in Table 6 show that PP, MP, and FI and the endurance indices during pre-supplementation were not significantly different between the groups. The results also reflect the fatigue effects of intense exercise on FI and endurance performance. The FI and endurance demonstrated significantly negative effects within the PG. In contrast to PG, the LG exhibited significant improvement in the MP, FI, and endurance indices compared with the PG. Individuals in the LG also maintained their performance between pre- and post-supplementation.

### 3.6. Effects of L. plantarum PS128 on Free Amino Acid Content after Supplementation

Table 7 lists the amino acid profiles between the groups after supplementation and the championship triathlon stimulation. The LG demonstrated significantly higher histidine, leucine, isoleucine, threonine, valine and glutamine values than the PG (*p* < 0.05).

## 4. Discussion

Triathlon athletes who were supplemented with *L. plantarum* PS128 probiotics over the long-term had significantly decreased CK content. However, other indices related to muscular injury (e.g., LDH, Protein carbonyl, Myoglobin) and fatigue (lactate, ammonia, FFA) remained unchanged after the triathlon competition. However, the pro- (TNF-α, IFN-γI, L-6, and IL-8) and anti- (IL-10) inflammation cytokines, kidney injury (TRX and C5a), and oxidative stress (MPO) markers induced by intense exercise were significantly improved with the *L. plantarum* PS128 intervention. The profiles of amino acid contents were significantly different from the placebo treatment. Branch chain amino acids (BCAAs), such as threonine, glutamine, and histidine, are likely modulated by probiotics supplementation. In addition, the athletes’ anaerobic and aerobic exercise capacities may have been compensated for by triathlon-induced fatigue; the athletes could have maintained their performance, compared with previous times. Therefore, *L. plantarum* PS128 may be a potential alternative ergogenic aid to improve the health of athletes and the general public.

The results of muscle injury and fatigue indices after the sprint triathlon and the triathlon championship are presented in Table 2 and Table 3. Previous studies have validated that intense exercise can cause muscle tissue damage, calcium ion imbalance, an infiltration of neutrophils, the production of free radicals, and the excretion of cytokines, resulting in oxidative injury, inflammation, and the accumulation of associated markers (CK and LDH) [21,22]. Nutrient supplementation, such as with whey protein and spinach, is also considered a protective strategy to mitigate intense exercise-induced tissue injury [23,24]. *L. plantarum* TWK10 also exhibited bioactivity on physiological modulations regarding lactate, ammonia, and CK immediately after acute exercise intervention [15]. In this study, we found the *L. plantarum* PS128 did not exert beneficial effects on fatigue biomarkers immediately after intense exercise. However, the CK during the recovery phase was significantly lower in the supplementation group. Fatigue indices, including lactate, ammonia, and FFA, are considered to derive from energy utilization and balance. Excessive accumulation of metabolites and energy imbalance can result in central and peripheral fatigue [25]. *L. plantarum* PS128 may be useful for inflammation or oxidative modulation, rather than energy regulation or harvesting.

Intense exercise may substantially increase oxygen consumption, concomitant with ROS production [26]. Furthermore, the acute muscle inflammation caused by intensive contractions during exercise could result in leukocyte infiltration and increased levels of inflammatory cytokines, such as TNF-α and IL-6 [27]. Injured cells are devoured by infiltrated macrophages, concomitant with the production of ROS and TNF-α [28], and an appropriate inflammation process is essential to rebuilding normal tissue function and adaptation by anti-inflammation modulation [29]. In previous related studies, both pro- (TNF-α, IFN-γ, and IL-6) and anti- (IL-10) inflammation cytokines were significantly elevated after a triathlon challenge [30,31]. In our results (Table 3), *L. plantarum* PS128 not only decreased inflammatory cytokines, but also elevated the production of anti-inflammatory cytokines immediately after a championship triathlon. In the recovery phase (the 3hRest point), *L. plantarum* PS128 also demonstrated a beneficial effect on inflammatory cytokines.

TRX is a small and versatile protein that functions as a free radical scavenger to eliminate hydrogen peroxide and protect cells from oxidative injury [32]. In addition, TRX is an important substance secreted by renal tubular epithelial cells when kidneys are damaged by ischemia/reperfusion. Therefore, the content of TRX may be used as an indicator of exercise-induced renal injury caused by ischemia and oxidative stress [33]. Complement 5a (C5a) is a multifunctional pro-inflammatory mediator that increases vascular permeability and promotes leukocyte migration to the inflamed site and produces active oxides. A previous study also demonstrated urine C5a was positively associated with the severity of kidney injury [34]. Furthermore, urine TRX was significantly increased in triathletes after a triathlon competition, possibly due to renal tubule injury cause by long-term endurance exercise for anti-oxidative purposes [35]. In the current results from the related indices (Figure 3), *L. plantarum* PS128 supplementation significantly modulated TRX and MPO levels by elevating anti-oxidative capacities. On the other hand, the pro-inflammatory mediator C5a may be also significantly regulated by *L. plantarum* PS128 mediated IL-10 production (Table 3).

Branch chain amino acids, including valine, leucine, and isoleucine, have been reported to play roles in fatigue amelioration in endurance exercise in previous studies [36]. Supplementation with BCAAs in endurance-trained human skeletal muscles may partially suppress exercise-induced expression of PGC-1a for the activation of ubiquitin proteasome signaling and also suppress mTORC1 inhibitor DDIT4 expression [37]. Furthermore, metabolome analysis also showed that BCAA catabolism in muscles is important for homeostasis of muscle energy metabolism and energy index for adaptation to exercise training [38]. In the current study, *L. plantarum* PS128 combined with regular training demonstrated a significant elevation of BCAAs content in the plasma sample. We confirmed that this result did not arise from the exogenous intake of food or other dietary supplements. Therefore, we believe that the metabolism of microbiota may have caused the difference.

In previous studies, exercise performance has been addressed for specific interventions or supplementations; however, few studies have focused on performance during the recovery phase—a situation that is possible in fatigue conditions. The FI is an indicator of power reduction during 30 seconds of power output. A higher FI index represents a higher fatigue condition after a higher workout load [39]. After a triathlon, a neuromuscular deficit arises due to impairments in force transmission, resulting in a lower-than-average positive force with a slower rate of force development [40]. We found that *L. plantarum* PS128 could maintain the mean power and FI compared with pre-test data. It is possible to ascribe the improvement in muscular adaptation to the effects of plasma metabolites and immune regulation described above. This finding may confer potential benefits to athletic training and intensity adjustment in the field of sports science.

## 5. Conclusions

The above study and the results of the current study validate how high-intensity exercise, such as participation in marathons and triathlons, can cause a series of inflammatory and oxidative injuries to physiological homeostasis. *L. plantarum* PS128 has been shown to have beneficial effects on exercise performance maintenance, made possible via modulation of inflammation, oxidation, and the metabolism. Actually, marathons and triathlons have been quite popular amongst amateurs for health promotion or a sense of achievement. Therefore, probiotics could be considered as an alternative option for nutritional supplementation, not only for performance, but also for physiological adaptation.

## Figures and Tables

**Table 1:** Characteristics of the triathletes.

	Study I		Study II	
	PG	LG	PG	LG
Age (year)				
Median	21.1 ± 1.5	20.2 ± 0.7	20.1 ± 0.3	22.3 ± 1.2
Range	19–24	19–21	19–21	19–26
Height (cm)	171 ± 5	168 ± 8	171 ± 1	174 ± 1
Weight (kg)	64.8 ± 5.7	63.5 ± 8.5	65.6 ± 2.0	68 ± 2.8
BMI (kg/m^2^)	22.1 ± 1.3	22.5 ± 1.2	22.4 ± 0.5	22.4 ± 0.9
VO_2_ max (mL/kg/min)	-	-	60.1 ± 2.2	61.8 ± 1.5
Calories (Kcal)	2002 ± 312	2250 ± 143	2079 ± 262	2048 ± 242

The data are represented as mean ± SEM in individual indexes with Study I (*n* = 18) and II (*n* = 16). The calories were assessed during the period of probiotics supplementation. PG and LG groups mean the placebo and *L. plantarum* treatment groups, respectively.

**Table 2:** Supplementation effects on fatigue and injury-related biochemical indices (Study I).

	Basal		AfterEx		3hRest	
	PG	LG	PG	LG	PG	LG
**Muscle damage**						
CK (U·L^−1^)	171.4 ± 46	175.3 ± 40	165.4 ± 32	155.9 ± 29	174.4 ± 34	160.5 ± 28
LDH (U·L^−1^)	149.4 ± 9	138.9 ± 8	257.0 ± 30 ^+^	194.3 ± 15 ^+^	209.0 ± 14^+^	185.2 ± 15 ^+^
Protein carbonyl (nmol·mL^−1^)	2.3 ± 0.2	2.5 ± 0.2	2.5 ± 0.1	2.5 ± 0.1	2.6 ± 0.1	2.6 ± 0.1
**Muscle fatigue**						
Ammonia (μmol·L^−1^)	153.6 ± 4	148.9 ± 5	196.6 ± 13 ^+^	191.2 ± 17 ^+^	131.6 ± 16 ^#^	134.6 ± 7.6 ^#^
Lactate (mmol·L^−1^)	2.6 ± 0.2	2.6 ± 0.2	7.0 ± 0.9 ^+^	5.9 ± 1.1 ^+^	2.1 ± 0.3 ^#^	2.0 ± 0.3 ^#^
FFA (μM)	134 ± 25	113 ± 17	360 ± 52 ^+^	340 ± 53 ^+^	278 ± 59 ^+^	250 ± 30 ^+^

The biochemical indices were collected and assessed from Study I with the sprint triathlon intervention and the sampling time points included Basal, AfterEx, and 3hRest. The data are represented as mean ± SEM. PG (*n* = 9) and LG (*n* = 9) indicate the placebo and *L. plantarum* PS128 supplementation, respectively. ^+^ indicates a significant difference compared with the basal point within groups; ^#^ indicates a significant difference compared with the AfterEx point within the groups. The significant difference within and between groups was considered when *p* < 0.016 (0.05/3).

**Table 3:** Supplementation effects on fatigue and injury-related biochemical indices (Study II).

	Basal		AfterEx		3hRest	
	PG	LG	PG	LG	PG	LG
**Muscle damage**						
CK (U L^−1^)	237.3 ± 45	220.1 ± 34	662.3 ± 22 ^+^	548.5 ± 99^+^	1866 ± 67 ^+^	806.0 ± 13 ^+,^*
LDH (U L^−1^)	242.8 ± 14	224.2 ± 8	266.3 ± 20	258.0 ± 19	309.8 ± 22 ^+^	270.0 ± 19 ^+^
Myoglobin (ng mL^−1^)	21.8 ± 2	21.3 ± 2	453.2 ± 144^+^	306.6 ± 47 ^+^	379.3 ± 134 ^+^	272.4 ± 30 ^+^
**Muscle fatigue**						
FFA (μM)	262 ± 49	254 ± 41	536 ± 106 ^+^	462 ± 75 ^+^	437 ± 59 ^+^	402 ± 72 ^+^

The biochemical indices were collected and assessed from Study II with the championship triathlon interventionm and the sampling time points included Basal, AfterEx, and 3hRest. The data are represented as mean ± SEM. PG (*n* = 9) and LG (*n* = 9) indicate the placebo and *L. plantarum* PS128 supplementation, respectively. * indicates a statistically significant difference between the groups; ^+^ indicates a significant difference compared with the basal point within the groups; ^#^ indicates a significant difference compared with the AfterEx point within the groups. A significant difference within and between groups was considered when *p* < 0.016 (0.05/3).

**Table 4:** Supplementation effects on the cytokine expression profile after exercise (Study I).

	Basal		AfterEx		3hRest	
	PG	LG	PG	LG	PG	LG
**Pro-inflammation**						
TNF-α (pg/mL)	11.4 ± 1.3	9.9 ± 0.8	14.4 ± 1.8 ^+^	10.5 ± 0.8 *	15.2 ± 1.9 ^+^	10.4 ± 0.7
IFN-γ (pg/mL)	12.3 ± 1.6	12.4 ± 2.2	12.4 ± 0.9 ^+^	13.3 ± 2.8 *	12.1 ± 1.2 ^+^	12.5 ± 2.1
IL-6 (pg/mL)	5.8 ± 0.4	5.2 ± 0.4	9.3 ± 1.2 ^+^	6.6 ± 0.7 *	7.6 ± 0.9 ^+^	5.5 ± 0.5 *
IL-8 (pg/mL)	5.4 ± 0.4	5.1 ± 0.4	6.9 ± 0.7 ^+^	5.0 ± 0.4 *	5.6 ± 0.4 ^+^	5.4 ± 0.6
**Anti-inflammation**						
IL-4 (pg/mL)	12.0 ± 2.5	11.5 ± 2.1	13.4 ± 3.3 ^+^	11.2 ± 2.0 ^+^	12.3 ± 3.0 ^+^	11.2 ± 2.0
IL-10 (pg/mL)	4.4 ± 0.3	3.7 ± 0.4	8.7 ± 1.1 ^+^	9.4 ± 1.9 ^+^	4.1 ± 0.4 ^#^	3.7 ± 0.3 ^#^

The cytokine profiles were collected and assessed from Study I with the sprint triathlon intervention, and the sampling time points included Basal, AfterEx, and 3hRest. The data are represented as mean ± SEM. PG (*n* = 9) and LG (*n* = 9) indicate the placebo and *L. plantarum* PS128 supplementation, respectively. * indicates a statistically significant difference between the groups; ^+^ indicates a significant difference compared with the basal point within the groups; ^#^ indicates a significant difference compared with the AfterEx point within the groups. A significant difference within and between groups was considered when *p* < 0.016 (0.05/3).

**Table 5:** Supplementation effects on the cytokine expression profile after exercise (Study II).

	Basal		AfterEx		3hRest	
	PG	LG	PG	LG	PG	LG
**Pro-inflammation**						
TNF-α (pg/mL)	13.9 ± 2.2	14.3 ± 1.7	22.1 ± 2.4 ^+^	15.2 ± 1.9 *	22.5 ± 3.0 ^+^	15.3 ± 2.2 *
IFN-γ (pg/mL)	2.4 ± 0.4	1.7 ± 0.1	2.8 ± 0.5	1.4 ± 0.1 *	2.8 ± 0.4	1.5 ± 0.1 *
IL-6 (pg/mL)	9.3 ± 1.2	7.9 ± 0.5	19.7 ± 2.2 ^+^	14.1 ± 1.3 *^,+^	11.6 ± 1.2 ^#^	8.0 ± 1.0 *^,#^
IL-8 (pg/mL)	104.8 ± 12.6	98.9 ± 3.2	121.3 ± 11	92.4 ± 7.4 *	126.1 ± 11.5	98.1 ± 6.8 *
**Anti-inflammation**						
IL-4 (pg/mL)	0.8 ± 0.1	0.8 ± 0.0	0.7 ± 0.0	0.7 ± 0.0	0.7 ± 0.0	0.7 ± 0.0
IL-10 (pg/mL)	10.5 ± 0.8	10.1 ± 0.6	84.6 ±1 8 ^+^	131.7 ± 14 *^,+^	13.3 ± 1.3 ^+,#^	13.4 ± 1.3 ^+,#^

The cytokine profiles were collected and assessed from Study II with the championship triathlon intervention, and the sampling time points included Basal, AfterEx, and 3hRest. The data are represented as mean ± SEM. PG (*n* = 8) and LG (*n* = 8) indicate the placebo and *L. plantarum* PS128 supplementation, respectively. * indicates a statistically significant difference between the groups; ^+^ indicates a significant difference compared with the basal point within the groups; ^#^ indicates a significant difference compared with the AfterEx point within the groups. A significant difference within and between groups was considered when *p* < 0.016 (0.05/3).

**Table 6:** Supplementation effects on Wingate and VO_2_ max test after the triathlon championship.

	Pre		Post	
	PG	LG	PG	LG
**Wingate**				
PP (W)	843.6 ± 57	891.5 ± 42	848.8 ± 60 ^+^	971.6 ± 28 *
MP (W)	631.5 ± 36	698.0 ± 28	633.0 ± 30 ^+^	710.4 ± 11 *
FI (%)	50.0 ± 2.2	45.2 ± 2.5	56.2 ± 1.7 ^+^	49.8 ± 2.5 *
**85 % VO_2_ max**				
Endurance (sec)	1608.3 ± 304	2141.5 ± 683	898.2 ± 151 ^+^	1997.5 ± 480 *

In Study II, the post-Wingate and endurance tests were conducted at 72 and 48 hours, respectively, after the championship triathlon. The data are represented as mean ± SEM, and the PP, MP, and FI correspond to the mean peak power, mean power, and fatigue index, respectively. * indicates a statistically significant difference between the groups, and + indicates a significant difference compared with the basal point within the groups. The significant difference within and between groups was considered when *p* < 0.05.

**Table 7:** Plasma concentrations of free amino acids after the supplementation.

	PG	LG	Normal (nmol/mL)
Alanine	465.8 ± 36	537.2 ± 69	184–590
Arginine	40.5 ± 4.5	39.4 ± 7.3	6.45–108
Asparagine	60.2 ± 2.7	65.6 ± 5.2	25–74
Aspartic acid	15.8 ± 2.0	13.6 ± 0.4	1.0–20
Cysteine	39.2 ± 2.0	44.5 ± 3.2	26–90
Glutamic acid	81.7 ± 3.6	87.7 ± 3.3	5–120
Glutamine	618 ± 34	653 ± 39	350–880
Glycine	269.0 ± 6	275.2 ± 25	125–340
Histidine	91.6 ± 2.6	105.6 ± 4.5 *	56–124
Isoleucine	63.2 ± 4.9	88.7 ± 9.1 *	37–110
Leucine	125.5 ± 6.4	212.8 ± 60 *	75–206
Lysine	167.4 ± 8.0	202.2 ± 25	93–336
Methionine	25.2 ± 1.8	30.3 ± 3.0	5–44
Phenylalanine	73.8 ± 2.9	81.5 ± 7.3	36–83
Proline	247.6 ± 21.4	253.7 ± 15.7	80–274
Serine	131.6 ± 5.4	140.3 ± 8.9	65–183
Threonine	137.9 ± 7.7	165.8 ± 9.4 *	80–246
Tryptophan	46.2 ± 6.4	39.4 ± 8.1	25–73
Tyrosine	75.6 ± 4.9	79.5 ± 3.2	43–99
Valine	257.8 ± 11	321.3 ± 20 *	127–367

In Study II, blood amino acid was assessed after three weeks’ supplementation and triathlon championship intervention. The data was represented as mean ± SEM and *, (*p* < 0.05) means the significant difference between groups. A normal range of amino acid existed in physiological concentrations.
